# Non-high-density lipoprotein cholesterol may predict the cardio-cerebrovascular risk in patients on maintenance hemodialysis

**DOI:** 10.1186/s12944-021-01546-1

**Published:** 2021-11-13

**Authors:** Denggui Luo, Yueming Luo, Yanhong Zou, Yuanzhao Xu, Bo Fu, Dong Yang, Jun Yang, Cai Xu, Shuyi Ling, Shunmin Li, Airong Qi

**Affiliations:** The Fourth Affiliated Hospital of Guangzhou University of Chinese Medicine, Shenzhen Traditional Chinese Medicine Hospital, Shenzhen, China

**Keywords:** Non-LDL-C, Cardio-cerebrovascular risk, Maintenance hemodialysis

## Abstract

**Background:**

Non-high-density lipoprotein cholesterol (non-HDL-C) may be an independent risk factor for cardio-cerebrovascular disease (CVD); however, the cutoff level in patients on maintenance hemodialysis (MHD) is unknown.

**Methods:**

This was a retrospective multicenter study of MHD patients treated at 10 dialysis centers in Guangdong Province from July 1, 2016, to April 1, 2017. Laboratory test data were collected and CVD complications and outcomes recorded.

**Results:**

In total, 1288 eligible patients were included in this study; the non-HDL-C interquartile range was 2.76 (2.24–3.45) mmol/L. Over a median follow-up time of 24 months, 141 patients developed CVD. The non-HDL-C level was a principal risk factor for such events (*P* < 0.05; 95% confidence interval 0.800–0.842). The maximum Youden index was 0.549 and the best cutoff > 3.39 mmol/L.

**Conclusion:**

Higher baseline non-HDL-C levels may increase the CVD risk in MHD patients. Thus, non-HDL-C effectively predicts CVD.

## Background

Chronic kidney disease (CKD) is associated with significant morbidity and mortality. In 2017, 1.2 million people worldwide died from CKD [1]. End-stage renal disease (ESRD) has become a major public health problem given increased life expectancies worldwide [2]. More than 2.5 million people are on renal replacement therapy; the number is projected to double by 2030 [3]. Such patients are at high risk of cardio-cerebrovascular disease (CVD), which independently predicts a need for dialysis [4, 5]. Attempts to reduce CVD in ESRD patients have usually been extensions of strategies employed for general populations [6]. Dyslipidemia in ESRD patients, and frequent changes in lipid and lipoprotein levels, greatly contribute to CVD development [7]. Certain dyslipidemia patterns increase the risk of atherosclerotic vascular disease in general populations. It thus seems likely that dyslipidemia increases the CVD risk in ESRD patients. Such dyslipidemia is characterized by high triglyceride (TG) and low high-density lipoprotein cholesterol (HDL-C) levels [8]. However, prior studies evaluating associations between specific lipid and lipoprotein levels and CKD were limited in terms of scope and generalizability [9]. Although some studies suggested no, or an inverse, association between low-density lipoprotein cholesterol (LDL-C) levels and the CVD risk in patients on maintenance hemodialysis (MHD), the effects of lipid levels remain unknown [10]. This was a multicenter cross-sectional study of 1876 dialysis patients. The trends, and the effects of confounding factors, were validated and adjusted by dividing patients into quartiles (1/4, 3/4). The study seeks to improve the definition and prevention of, and therapy for, dyslipidemia in dialysis patients.

## Methods

### Study design and participants

This retrospective study was conducted at 10 hospitals in southern Guangdong Province. All hospital laboratories complied with the Guangdong Standard Operation Procedure for Blood Purification and had passed the quality and ability tests of the Guangdong Medical Association [11].

**Fig. 1:**
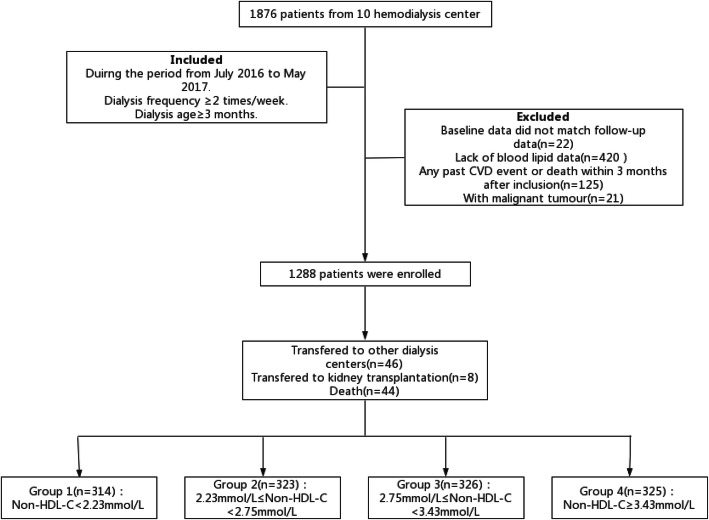
Flow chart of the participants in the study cohort

Demographic and dialysis-related data were collected using Epidata Entry ver. 3.1.1203.2006. The study included 1876 patients who underwent regular hemodialysis from July 2016 to July 2017. Additional inclusion criteria were: (1) at least 2 dialysis days/ week, and (2) dialysis duration ≥3 months. The exclusion criteria were: (1) missing baseline or follow-up data (especially lipid data), (2) any past CVD event or death within 3 months after inclusion, and/or (3) a tumor (Fig. 1).

### Biochemical analysis

Serum samples were drawn at MHD commencement and analyzed locally. For all patients, the fasting plasma glucose, serum albumin, hemoglobin, potassium, total cholesterol (TC), HDL-C, TG, LDL-C, blood urea nitrogen, uric acid, white blood cell, platelet, creatinine, calcium, phosphate, and parathyroid hormone (PTH) levels, and the parameter Kt/V (a measure of the efficacy of hemodialysis), were measured at regular but different intervals. The body mass index (BMI) for each patient was calculated as the post-dialysis body weight divided by the square of the height.

### Data collection and measures

General information, medical histories, and laboratory data were collected by physicians. All CVD complications were diagnosed by specialists at each center by reference to the symptoms and signs at onset, and laboratory and imaging data; the latter included coronary angiography, brain computed tomography (CT), and brain magnetic resonance imaging (MRI). The data were stored in Excel format. The study adhered to the principles of the Declaration of Helsinki. The work was approved by the Human Research Ethics Committees of the involved hospitals. Investigators or persons authorized by the investigators explained the benefits and risks of trial participation to each patient, or their legal representatives or notaries. Trial data were stored in a safe in the office of the first author, who performed all statistical analyses.

### Baseline and outcome data

Patient sex, age, and independence (or not) were recorded. Outcomes were assessed from baseline until discharge (i.e., the end of intervention) (Table 1). The primary outcomes were CVDs, including myocardial infarction, acute left heart failure, non-myocardial acute coronary syndrome, cerebral infarction, and cerebral hemorrhage. The pre-specified secondary outcomes were the association of the non-HDL-C level with CVD and the predictive utilities of the LDL-C, TC, and non-HDL-C levels.

**Table 1:** Baseline MHD patient characteristics according to Non-HDL-C quartile

Characteristic	Non-HDL-C Quartile				***p***-value
	Q1 < 2.24	2.24 ≤ Q2<2.76	2.76 ≤ Q3<3.45	Q4 ≥ 3.45	
	*n* = 314	*n* = 323	*n* = 326	*n* = 325	
Age (years)	57.89 ± 14.17	59.84 ± 15.09	59.47 ± 15.51	62.86 ± 15.07	< 0.001
Gender					0.469
male, n (%)	185(58.9%)	194(60.1%)	184(56.4%)	203(62.5%)	
female, n (%)	129(41.1%)	129(39.9%)	142(43.6%)	122(37.5%)	
Dialysis time (months)	28.11(14.09–65.67)	25.17(11.56–53.33)	23.90(9.50–50.40)	26.47(7.99–47.01)	
BMI (kg/m2)	19.71(14.50–25.00)	19.71(0.00–23.55)	19.59(0.00–24.72)	22.52(16.51–27.77)	< 0.001
Cause of ESRD, n (%)					< 0.001
primary glomerulonephritis	177(56.4%)	161(50.0%)	164(50.5%)	120(36.9%)	
diabetic nephropathy	65(20.7%)	68(21.1%)	68(20.9%)	87(26.8%)	
hypertensive nephropathy	19(6.1%)	12(3.7%)	12(3.7%)	18(5.5%)	
Anticoagulant type, n (%)					0.136
low molecular weight heparin	282(89.8%)	274(84.8%)	276(84.9%)	271(83.4%)	
unfractionated heparin	30(9.6%)	42(13.0%)	41(12.6%)	42(12.9%)	
Dialysis vascular access, n (%)					0.033
AVF	268(85.4%)	277(85.8%)	278(85.3%)	262(80.6%)	
AVG	3(1.0%)	2(0.6%)	6(1.8%)	6(1.8%)	
TCC	30(9.6%)	37(11.5%)	24(7.4%)	31(9.5%)	
NCC	13(4.1%)	7(2.2%)	18(5.5%)	26(8.0%)	
Mean UFV(L)	2.43(1.93–3.00)	2.40(1.65–2.93)	2.23(1.66–2.84)	2.30(1.71–3.16)	0.788
Systolic pressure (mmHg)	146.35 ± 18.99	145.72 ± 21.37	146.71 ± 20.86	145.37 ± 20.77	0.838
Diastolic pressure (mmHg)	82.84 ± 11.89	82.50 ± 12.49	84.68 ± 50.09	84.17 ± 40.21	0.814
Diabetes, n (%)	59(18.8%)	63(19.5%)	77(23.7%)	71(21.8%)	0.404
FPG (mmol/L)	4.35 ± 1.81	6.99 ± 3.02	4.86 ± 1.25	7.42 ± 2.84	0.058
Tc (mmol/L)	4.40 ± 1.28	3.87 ± 0.42	5.20 ± 1.25	5.32 ± 0.24	< 0.001
Tg (mmol/L)	3.36 ± 2.85	0.81 ± 0.25	1.67 ± 1.55	3.09 ± 2.75	< 0.001
LDL-C (mmol/L)	2.88(2.40–3.70)	2.06(1.83–2.33)	1.69(1.39–2.66)	2.02(1.54–2.41)	< 0.001
HDL-C (mmol/L)	1.14(0.92–1.44)	1.08(0.88–1.27)	1.07(0.88–1.34)	1.07(0.85–1.42)	0.052
Alb (g/L)	22.04 ± 17.71	42.75 ± 1.89	31.74 ± 16.65	37.75 ± 3.09	< 0.001
Cr (μmol/L)	554.36 ± 563.53	1082.75 ± 161.16	937.10 ± 654.46	1194.25 ± 307.89	< 0.001
BUN (μmol/L)	27.18(22.64–34.00)	27.40(22.80–33.90)	26.18(22.18–32.43)	26.97(22.00–34.12)	0.638
Ua (μmol/L)	266.60 ± 211.82	458.75 ± 35.85	465.30 ± 271.41	547.25 ± 133.98	< 0.001
Total Kt/V	1.33(1.12–1.60)	1.32(1.18–1.50)	1.32(1.18–1.48)	1.37(1.12–1.76)	0.029
WBC (10^9^/L)	4.72 ± 1.44	6.40 ± 1.45	6.15 ± 2.53	7.00 ± 1.31	0.035
Hb (g/L)	128.27 ± 34.91	99.00 ± 12.02	107.00 ± 41.59	90.25 ± 26.53	< 0.001
Plt (10^9^/L)	131.90 ± 51.11	23.17 ± 2.92	30.83 ± 22.22	24.84 ± 4.37	< 0.001
K (mmol/L)	5.31(4.62–6.40)	5.15(4.50–5.70)	5.05(4.60–5.62)	5.40(4.63–7.00)	0.084
Ca (mmol/L)	2.20(2.08–2.36)	2.16(2.00–2.32)	2.15(2.04–2.31)	2.31(2.12–2.76)	0.012
PTH (pg/ml)	167.60(38.65–403.60)	260.96(75.05–585.33)	234.41(72.90–624.25)	233.93(79.03–587.40)	0.009
P (mmol/L)	6.80 ± 5.89	1.79 ± 0.47	3.56 ± 2.83	2.46 ± 0.44	< 0.001

Values are expressed as mean ± SD, median and interquartile range, or number (percentage) as appropriate. *BMI* Body mass index, *ESRD* End-stage renal disease, *AVF* Autogenous arteriovenous fistula, *AVG* Arteriovenous graft, *TCC* Tunnel-cuffed catheter, *NCC* Non-cuffed catheter, *UFV* Ultrafiltration volume, *CVD* Cardiovascular disease, *FPG* Fasting plasma glucose, *Tc* Total cholesterol, *Tg* Triglycerides, *LDL-c* Low density lipoprotein cholesterol, *HDL-C* High-density lipoprotein cholesterol, *Alb* Albumin, *Cr* Creatinine, *BUN* Blood urea nitrogen, *Ua* Uric acid, *Hb* Hemoglobin, *Plt* Platelets, *Pth* Parathyroid hormone

### Statistical analyses

All patients were divided into four groups by reference to the baseline non-HDL-C quartiles. SPSS software (Version 25.0; Chicago, IL, USA) and MedCalc software (Version 16.8; Ostend, Belgium) were used for all analyses. Continuous variables that were normally distributed are given as^−^x ± *s* and a one-way ANOVA was used for comparisons. Continuous variables that were not normally distributed are shown as medians (quartiles 1, 3) and compared with the aid of the Kruskal-Wallis test. Categorical variables are expressed as frequencies (proportions) and were compared using the chi-squared test. The cumulative survival rate was calculated by the Kaplan-Meier method; survival curves were plotted. The log-rank test was used to test significance. The relationship between the non-HDL-C level and CVDs was analyzed via Cox’s proportional hazard regression and the results expressed as hazard ratios (*HR*s) with 95% confidence intervals (CIs). The area under the receiver operator curve (AUC) and the 95% CI were used to evaluate the predictive utility of the non-HDL-C level in terms of various endpoints. The AUC ranged from 0.5 (indicating randomness) to 1.0 (complete dependence). The maximum Youden index was used to determine the optimal non-HDL-C cutoff for each endpoint. A two-sided α ≤ 0.05 was taken to indicate significance.

## Results

### Participant characteristics

**Fig. 2:**
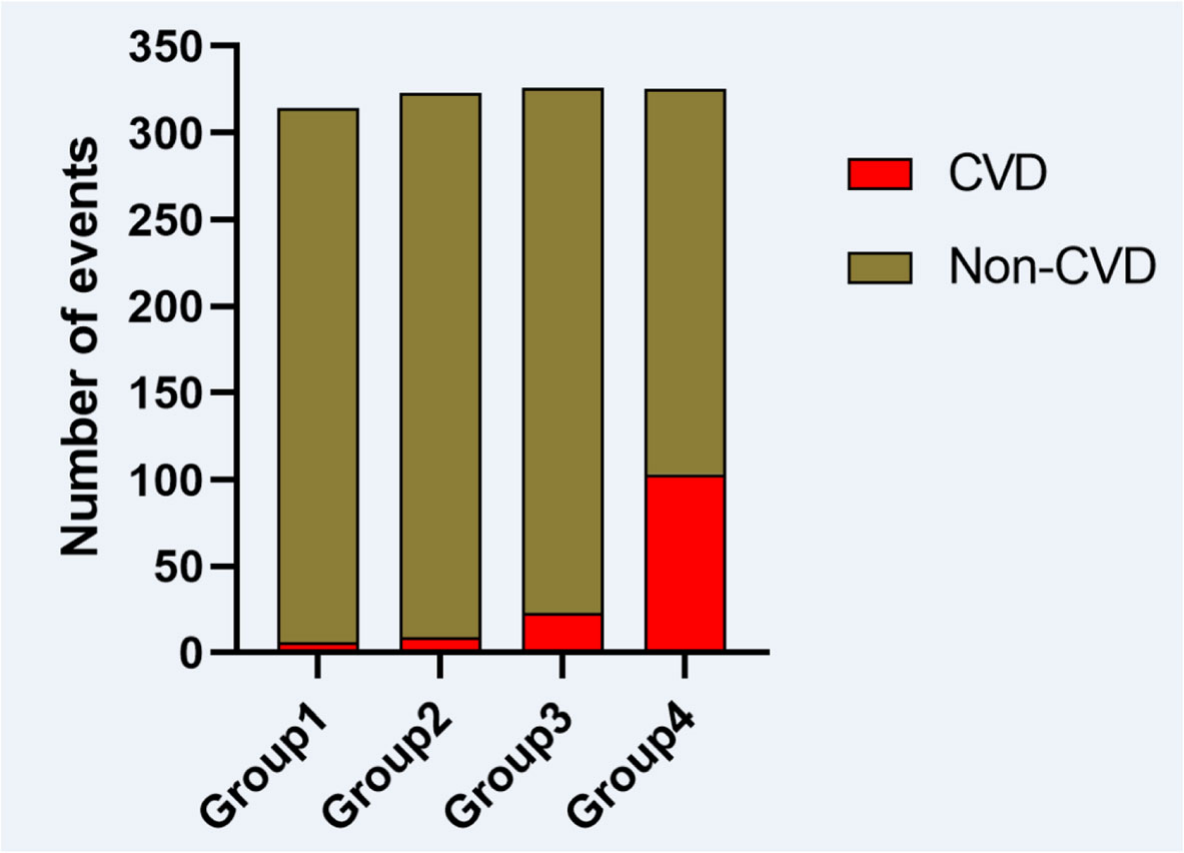
The comparison of CVD events between each group

**Fig. 3:**
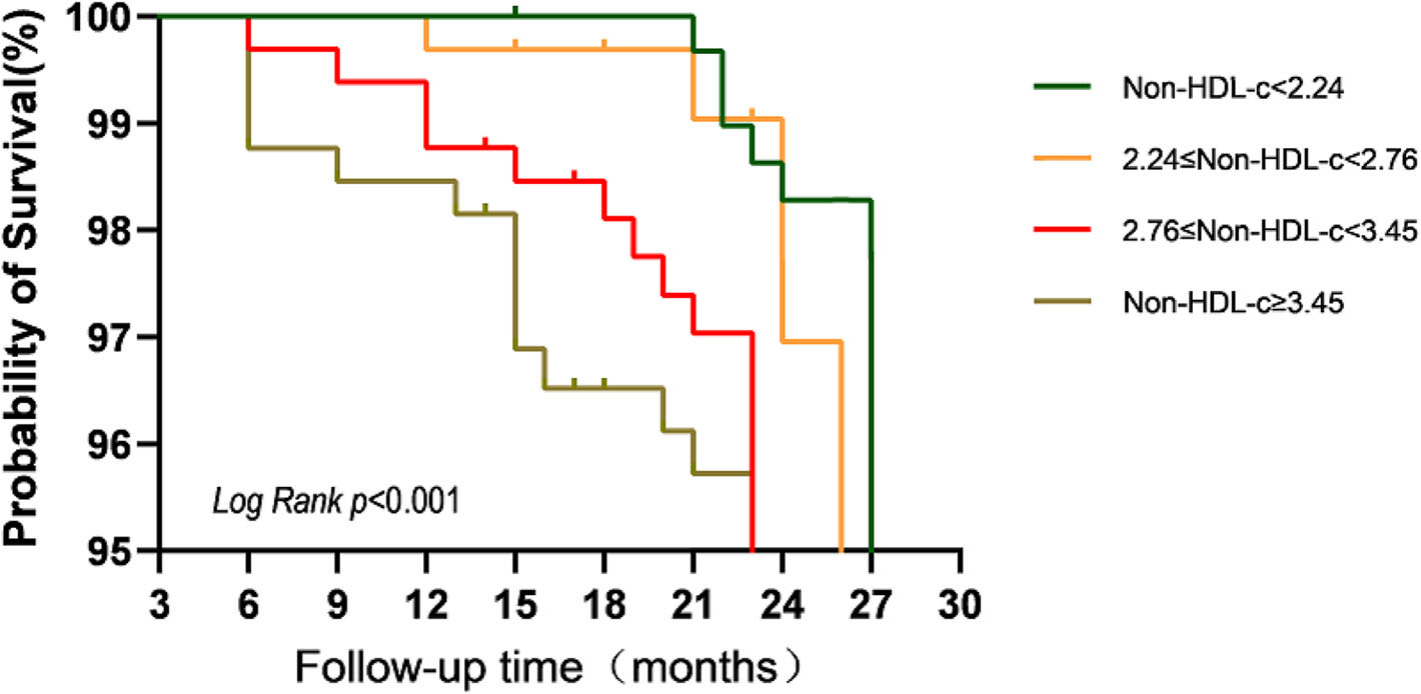
Kaplan–Meier curves of MHD patients with different levels of Non-HDL-C cardiovascular mortality

**Fig. 4:**
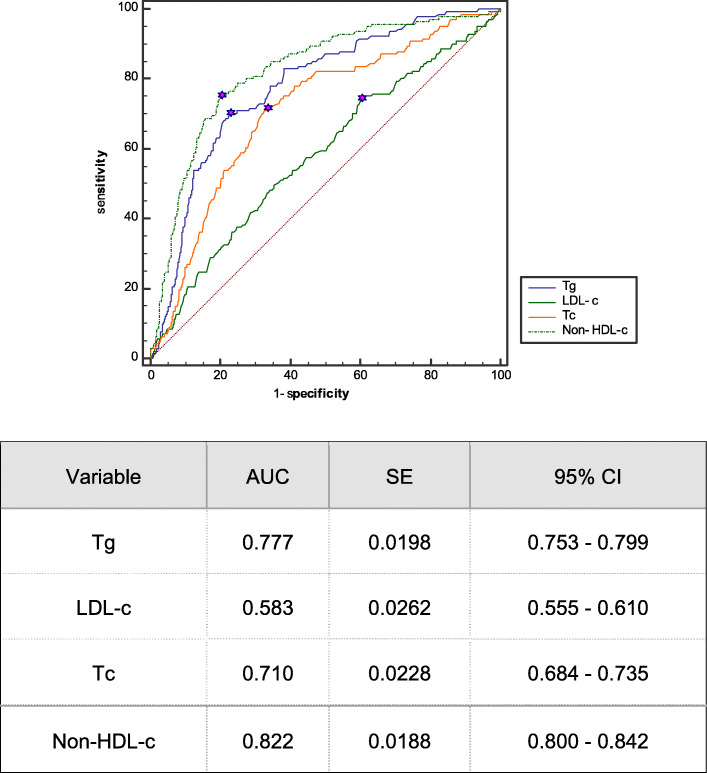
ROC curve chart of the predictive effect of LDL-C, TC and Non-HDL-C on CVD events in MHD patients

Ultimately, a total of 1288 patients aged 59.85 ± 15.06 years were enrolled; 766 males (59.5%) and 522 females (40.5%). The non-HDL-C interquartile range was 2.76 (2.24–3.45) mmol/L. Age; pre-dialysis weight; and the levels of white and red blood cells and platelets, serum creatinine and albumin, ferritin, and blood calcium differed among the four quartiles (all *P* < 0.05). The median follow-up time was 24 months. During this time, 141 (10.94%) patients experienced CVD for the first time, including 39 (3.02%) with acute myocardial infarctions, 32 (2.48%) with cerebral infarctions, 55 (4.27%) with intracerebral hemorrhages, and 15 (1.16%) with acute coronary syndromes (Table 2). Patients in the non-HDL-C ≥ 3.45 mmol/L group exhibited a higher CVD rate (31.7%) than the other groups (Fig. 2). Univariate Cox’s regression showed that age and the levels of white blood cells, platelets, blood glucose, TC, TG, non-HDL-C, and total blood protein were risk factors for CVD (all *P* < 0.05, Tables 3 and 4). The Kaplan-Meier survival curve revealed a positive correlation between the non-HDL-C level and CVD incidence (*P* < 0.01; Fig. 3). The ROC curves suggested that, compared to the TC (AUC 0.710, 95% CI 0.684–0.735), TG (AUC 0.777, 95% CI 0.753–0.799), and LDL-C (AUC 0.583, 95% CI 0.753–0.799) levels, the non-HDL-C level (AUC 0.822, 95% CI 0.800–0.842) better predicted CVD (Fig. 4). The maximum Youden index was 0.549, and the corresponding non-HDL-C cutoff 3.39 mmol/L. Next, the non-HDL-C level was included in a Cox regression using the quartiles as categorical variables. Single-factor regression showed that, after adjusting for age and sex using the Q1 group as a reference, Q2 (*P* < 0.01), Q3 (*P* < 0.01), and Q4 (*P* < 0.01) were at higher risks of CVD; the risks were not affected by diabetes status, dialysis duration, BMI, anticoagulant type, or systolic or diastolic blood pressure. After further adjustment for hemoglobin, serum albumin, and blood uric acid and creatinine levels, the Kt/V, average ultrafiltration rate, and platelet and serum urea nitrogen levels, the risk proportions remained different (and statistically significant) (*P* < 0.01, Table 4). Therefore, the non-HDL-C level was associated with an increased risk of cardiovascular disease in MHD patients.

**Table 2:** New-onset CVD events during a median 24 months follow-up in patients undergoing haemodialysis stratified according to Non-HDL-C quartile

Event	Patient groups based on Non-HDL-C levels				
	Q1 < 2.24	2.24 ≤ Q2<2.76	2.76 ≤ Q3<3.45	Q4 ≥ 3.45	
	*n* = 314	*n* = 323	*n* = 326	*n* = 325	*p*-value
Total CVD events	6(4.3%)	9(6.4%)	23(16.3%)	103(73.0%)	0.029
Myocardial infarction	0(0.0%)	1(0.7%)	6(4.3%)	32(22.7%)	
Cerebral infarction	3(2.1%)	5(3.5%)	8(5.7%)	16(11.3%)	
Cerebral haemorrhage	1(0.7%)	2(1.4%)	7(5.0%)	45(31.9%)	
Acute coronary syndrome	2(1.4%)	1(0.7%)	2(1.4%)	10(7.1%)	

Data presented as n of patients (%)

χ2-test was used for the comparison among the four groups

**Table 3:** The association of Non-HDL-C with CVD in the study cohort

Variable	Univariate analysis		Multivariate analysis	
	*HR(95%CI)*	*P-value*	*HR(95%CI)*	*P-value*
Age	1.022(1.011–1.034)	<0.001	1.020(1.009–1.032)	< 0.001
Gender	0.938(0.665–1.323)	0.714		
BMI	0.992(0.981–1.004)	0.196		
Total KT/V	0.989(0.977–1.002)	0.098		
WBC	1.078(1.044–1.114)	< 0.001	1.083(1.040–1.128)	< 0.001
Plt	1.005(1.003–1.007)	< 0.001	1.005(1.002–1.007)	< 0.001
Hb	1.000(0.997–1.004)	0.952		
FPG	1.037(1.007–1.067)	0.015	1.026(0.999–1.054)	0.061
K	0.996(0.989–1.003)	0.996		
Ca	0.996(0.989–1.003)	0.278		
P	0.983(0.912–1.060)	0.664		
Ua	1.001(1.000–1.002)	0.057		
Cr	1.000(1.000–1.001)	0.303		
Alb	1.008(0.990–1.026)	0.380		
Tc	1.053(1.020–1.087)	0.002		
Tg	1.080(1.049–1.111)	< 0.001	1.094(1.056–1.133)	< 0.001
LDL-c	0.996(0.991–1.002)	0.194		
HDL-c	0.068(0.038–0.120)	< 0.001		
Non-HDL-c	1.092(1.061–1.124)	< 0.001	1.124(1.087–1.161)	< 0.001
Diabetes	1.048(0.702–1.563)	0.819

**Table 4:** Cox regression analysis of different levels of Non-HDL-C and cardiovascular and cerebrovascular events

Variable	Model 1		Model 2		Model 3	
	*HR (95%CI)*	*P-value*	*HR (95%CI)*	*P-value*	*HR (95%CI)*	*P-value*
Continuous variable						
Non-HDL-C	1.102(1.070–1.134)	<0.001	1.135(1.094–1.177)	<0.001	1.266(1.020–1.572)	0.033
Categorical variables						
Q1	Reference		Reference		Reference	
Q2	0.041(0.018–0.094)	<0.001	0.036(0.016–0.084)	< 0.001	0.023(0.003–0.176)	< 0.001
Q3	0.070(0.035–0.138)	<0.001	0.060(0.030–0.119)	< 0.001	0.056(0.013–0.246)	< 0.001
Q4	0.224(0.142–0.354)	<0.001	0.175(0.109–0.282)	< 0.001	0.238(0.104–0.545)	0.001

Model 1: Adjusted for age and gender

Model 2: Adjusted for model 1 covariates and diabetes, dialysis time, BMI, anticoagulant type, systolic blood pressure and diastolic pressure

Model 3: Adjusted for model 2 covariates and hemoglobin, serum albumin, uric acid, serum Cr, Kt/V, Mean UFV, Platelets, Blood urea nitrogen

All variables with a confirmed *P*-value < 0.05

## Discussion

Hypercholesterolemia is an independent risk factor for coronary heart disease (CHD) and LDL-C is the principal laboratory parameter used for CVD management [12]. The experts of the National Lipid Association concluded that increased non-HDL-C and LDL-C levels were the root causes of atherosclerosis because they are involved in the majority of clinical CHD events [13, 14]. To reduce the risk of ischemic events in patients with CHD, the fasting LDL-C level should be controlled to < 1.4 mmol/L (primary goal) and the non-HDL-C level to < 2.2 mmol/L (secondary goal), according to the 2019 European Guide for the Year [15].

Serum LDL-C, TG, HDL-C, and non-HDL-C levels are associated with the risk of atherosclerotic CVD and other CV events [13, 14]. Serum β-trace protein and β2-microglobulin, and a composite of these markers with the eGFRcr and eGFRcys rates, were also independently associated with the risk of ESRD and all-cause mortality [16].

The non-HDL-C level is obtained by subtracting the HDL-C level from the TC level, and serves as a comprehensive indicator of the level of atherosclerotic lipids, including LDL-C, lipoprotein A (ApoA), intermediate-density lipoprotein (IDL), and very low-density lipoprotein (VLDL) remnants [17]; and as a marker of cardiovascular risk [18]. In 2018, the global age-standardized mean non-HD-C level was 3.3 mmol/L (range 3.2–3.4 mmol/L) for women and 3.3 mmol/L (range 3.3–3.4 mmol/L) for men [19], but the figures for dialysis patients remain unclear.

Over a median follow-up of 24 months, 141 patients suffered from CVD. Univariate Cox’s regression showed that age; anticoagulant type; and white blood cell, platelet, blood sugar, TC, TG, non-HDL-C, and total blood protein levels were risk factors for CVD (all *P* < 0.05). The Kaplan-Meier survival curve revealed a positive correlation between the non-HDL-C level and CVD incidence. The ROC curves suggest that, relative to TC, TG, LDL-C, and other indicators, non-HDL-C better predicted CVD in MHD patients. The Youden index maximum was 0.549, corresponding to a non-HDL-C cutoff of 3.39 mmol/L.

Compared to overseas large-scale studies [20], the LDL-C reductions that we observed were greater. Takahiro [21] found that the non-HDL-C levels predicted mortality and was minimally affected by the fasting or serum TG level. Meta-analyses and large prospective studies found that non-HDL-C levels at treatment were better predictors of CVD than the LDL-C levels [22]. The non-HDL-C level is a simple predictor of risk in patients using or discontinuing statins; there is no need for a fasting blood sample [23]. When post-prandial LDL-C and non-HDL-C goals were reassessed using the non-fasting cut-off points, the percentage attainments did not differ in the fasting and non-fasting states. It has been suggested that the control of non-HDL-C levels of afforded better clinical benefits than those delivered by the control of LDL-C levels [24]. Non-HDL-C assessment is better than LDL-C evaluation when exploring the percentage attainments of non-fasting lipid levels that improve the coronary health of dialysis patients [25]. Cesaro et al. [26] found that ApoA was an independent risk factor for CVD events, but clinical verification is lacking. Unfortunately, ApoA data were lacking in this study; such data are required in future studies.

### Study strengths and limitations

The strengths of the study include the large sample size and the involvement of 10 provincial dialysis centers; this enhances the generalizability of the findings. Also, all researchers strictly followed standard operating procedures. Transdermal dialysis was simple, associated with good patient acceptance. The dropout rate was only 3.5% and the exit rate 4.0%. The principal limitation is that the retrospective design may be associated with observer and/or performance bias; also, the follow-up time was short. A long-term, multi-center prospective study is required.

## Conclusions

In conclusion, this study found that the serum non-HDL-C levels correlated positively with the cardiovascular disease risk. Compared to the TG, TC, and LDL-C levels, the non-HDL-C level better predicted CVD events in MHD patients, and can thus serve as a new clinical marker. Physicians should closely monitor non-HDL-C levels to reduce CVD events in MHD patients.

## Data Availability

The datasets used and/or analysed during the current study are available from the corresponding author on reasonable request.
